# Network pharmacology: towards the artificial intelligence-based precision traditional Chinese medicine

**DOI:** 10.1093/bib/bbad518

**Published:** 2024-01-09

**Authors:** Peng Zhang, Dingfan Zhang, Wuai Zhou, Lan Wang, Boyang Wang, Tingyu Zhang, Shao Li

**Affiliations:** Institute for TCM-X, MOE Key Laboratory of Bioinformatics/Bioinformatics Division, BNRIST, Department of Automation, Tsinghua University, Beijing 100084, China; Institute for TCM-X, MOE Key Laboratory of Bioinformatics/Bioinformatics Division, BNRIST, Department of Automation, Tsinghua University, Beijing 100084, China; China Mobile Information System Integration Co., Ltd, Beijing 100032, China; Institute for TCM-X, MOE Key Laboratory of Bioinformatics/Bioinformatics Division, BNRIST, Department of Automation, Tsinghua University, Beijing 100084, China; Institute for TCM-X, MOE Key Laboratory of Bioinformatics/Bioinformatics Division, BNRIST, Department of Automation, Tsinghua University, Beijing 100084, China; Institute for TCM-X, MOE Key Laboratory of Bioinformatics/Bioinformatics Division, BNRIST, Department of Automation, Tsinghua University, Beijing 100084, China; Institute for TCM-X, MOE Key Laboratory of Bioinformatics/Bioinformatics Division, BNRIST, Department of Automation, Tsinghua University, Beijing 100084, China

**Keywords:** network pharmacology, traditional Chinese medicine, network target, artificial intelligence, deep learning

## Abstract

Network pharmacology (NP) provides a new methodological perspective for understanding traditional medicine from a holistic perspective, giving rise to frontiers such as traditional Chinese medicine network pharmacology (TCM-NP). With the development of artificial intelligence (AI) technology, it is key for NP to develop network-based AI methods to reveal the treatment mechanism of complex diseases from massive omics data. In this review, focusing on the TCM-NP, we summarize involved AI methods into three categories: network relationship mining, network target positioning and network target navigating, and present the typical application of TCM-NP in uncovering biological basis and clinical value of Cold/Hot syndromes. Collectively, our review provides researchers with an innovative overview of the methodological progress of NP and its application in TCM from the AI perspective.

## ORIGIN AND DEVELOPMENT OF TCM-NP

Traditional Chinese medicine (TCM) embodies the integration of clinical practice experience and theory of the Chinese nation in the prevention and treatment of diseases for thousands of years. TCM regards the human body as a complex system, and is characterized by its holistic perspective and treatment based on syndromes differentiation [1]. The major challenges for TCM maintain as how to uncover the biological basis of the human complex system, how to further elucidate various TCM terms, including syndromes and herbs/formulas, and their relationships, and how to derive new findings to facilitate precision medicine according to TCM rationales. Consistent with the holistic perspective of TCM, with the rise of interdisciplinary subjects such as bioinformatics, system biology and computational biology, the research paradigm of modern medicine has gradually shifted from ‘reductionism’ to ‘holism’, and the research strategy of disease diagnosis and treatment has also shifted from ‘single disease, single target and single drug’ to ‘multi-target and systematic regulation’, emphasizing the importance of analyzing the mechanism of complex diseases from the perspective of systems biology [2, 3]. In this context, TCM-NP arose as the crystallization of cross-innovation between traditional and modern medicine, information science and system science [4, 5]. TCM-NP-related hypotheses and ideas were proposed as early as in 1999 [6], and further developed in 2002 [7], earlier than the concept of ‘network pharmacology’ proposed in 2007 [8]. The characteristics of TCM-NP are highly consistent with the holism of TCM and the principle of treatment based on syndrome differentiation, which has become a frontier and hot spot in the field of traditional medicine research [4, 5, 9].

The emergence and development of artificial intelligence (AI) and multi-omics sequencing technologies provide a new bonding point and support for TCM-NP in promoting the precision TCM. From the methodological perspective, the key issue of NP is to transform the machine learning (ML) paradigm that focuses on object’s features into that of learning relationships among features, which could increase the object's feature space to realize feature enhancement [10]. In this regard, AI methods, especially deep learning-based ones, have made great strides in automatically learning feature representations from biomedical data and thus are playing an increasingly important role in the field of TCM-NP [11]. According to the Web of Science (WOS) statistics, as the rapid development of AI technology, the number of TCM-NP-related studies has increased steadily and rapidly, which has doubled since 2017. In particular, with the release of the first monograph [5] and standard [4], TCM-NP has ushered in new development opportunities in the era of AI and big data [12].

In this review, we will summarize TCM-NP-involved AI methods into three categories: network relationship mining, network target positioning and network target navigating, where representative methodological cases and state-of-the-art (SOTA)AI methods are introduced. We also present the typical applications of TCM-NP in Cold/Hot syndromes (a.k.a. TCM ZHENG), two basic concepts of TCM, for dissecting network relationships and their clinical value among syndromes, diseases and TCM formulas.

## METHODOLOGIES OF TCM-NP

### Network target concept and theory

**Figure 1:**
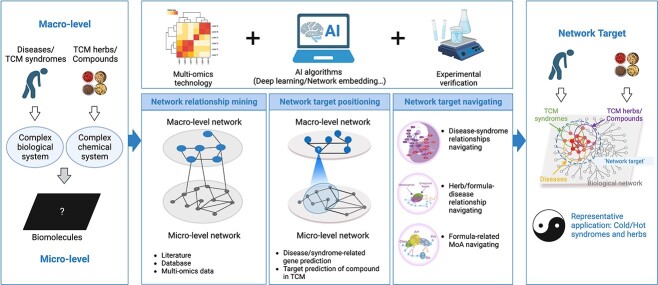
**Network target concept and key methodological framework for TCM-NP.** The network target emphasizes that the relationships between TCM herbs/compounds and TCM syndromes/diseases are reflected in the regulatory effects of TCM herbs/compounds on disease-related molecular network modules. From a methodological perspective, AI-based identification of potential network target can be categorized into ‘network relationship mining’, ‘network target positioning’ and ‘network target navigating’.

The main goal of the precision TCM is to reveal the mechanisms of action (MoA) of TCM formulas/drugs on diseases/syndromes, which could be briefly viewed as dissecting the relationships between diseases/syndromes and drugs/formulas [12]. From the perspective of TCM-NP, this goal involves systematically understanding the multilevel relationships between macro-level objects, including diseases/syndromes, traditional Chinese and Western drugs, and micro-level ones, including cells, genes and proteins, and identifying the network elements that can systematically couple these macro- and micro-level objects. This is in line with the focus of NP [8] and network medicine [13]. In this regard, the concept and theory of ‘network target’, proposed by Shao Li, is considered one of the earliest and most representative theories for understanding the MoA of TCM from the holistic perspective. This theory characterizes the main tasks of TCM-NP research: to construct disease or syndrome-related biological networks; to further infer the key modules of TCM formulas or components for intervening diseases or syndromes based on the biological network, as shown in Figure 1.

From the methodological perspective, AI-based identification of potential ‘network target’ that characterizes the relationships between diseases/syndromes and drugs/formulas can be categorized into three categories: (i) AI-based mining of relationships between macro- and/or micro-level objects from prior knowledge or omics data, which can be referred as ‘network relationship mining’ and viewed as the basis of TCM-NP methodologies. This issue could find echoes of natural language processing (NLP) tasks and network embedding tasks; (ii) AI-based new network relationships inference, which can be referred to as ‘network target positioning’. This tissue mainly includes ‘disease/syndromes–gene’ relationships prediction and ‘herb/natural products–target’ relationships prediction. (iii) AI-based identification of the network modules (i.e. network targets) connecting TCM drugs/formulas with diseases/syndromes based on the inferred network relationships, which referred to ‘network target navigating’. Examples of network navigating methods include disease–disease relationships analysis for syndrome differentiation, disease-drug relationships analysis for drug repurposing/discovery and formula recommendation, and drug–drug relationships analysis for drug synergistic effects and MoA of formulas. Of note, these above methods have been integrated into the UNIQ system (**U**sing **N**etwork target for **I**ntelligent and **Q**uantitative analysis on drug actions, UNIQ), the AI-based R&D platform our group has established to develop various new drugs [14, 15, 16, 17]. In the following three sections, we will provide an overview of the progress of AI-based methods involved in TCM-NP, where we also emphasize the role of SOTA AI-based methods, including graph neural networks (GNNs) and network embedding, summarized as Table 1.

**Table 1:** Representative AI algorithms/data resources involved in TCM-NP

TCM-NP methodologies	Categories	AI algorithms/data resources	Brief description	Publish year	Ref.
Network relationship mining	Prior knowledge-derived	KEGG	Relationships among genes and genomes	1999	[32]
		STRING	Relationships among proteins	2003	[31]
		TCMGeneDIT	Relationships among herbs, genes and diseases	2008	[37]
		DrugBank	Relationships between drugs and targets	2008	[39]
		PubChem	Relationships among small molecules	2009	[24]
		HIT	Relationships between herbal active ingredients and targets	2010	[36]
		DrugCentral	Relationships between drugs and targets	2016	[38]
		ChEMBL	Relationships among bioactive molecules with drug-like properties	2019	[25]
		ETCM	Relationships among herbs contained in formulas and their properties	2019	[28]
		SymMap	Relationships among TCM-related symptoms	2019	[34]
		DrugCombDB	Relationships among drugs	2020	[27]
		HERB	Relationships among herbs contained in formulas and their properties	2021	[29]
		BioGRID	Relationships among proteins	2021	[33]
		CDCDB	Relationships among drugs	2022	[26]
		TCMBank	Relationships among herbs contained in formulas and their properties	2023	[30]
	Omics data-derived	LMMA	Refined gene network combined literature and micro-array gene-expression data	2006	[49]
		Regression	Biological network of Hot syndrome and Cold syndrome	2013	[40]
		Bayesian inference	Multicellular function and disease with human tissue-specific networks	2015	[50]
		Single-cell Transcriptomics	Single-cell transcriptome network underlying gastric premalignant lesions and early gastric cancer	2019	[44]
		Nonlinear ordinary differential equations/ regulatory factors	Gene regulatory or signal transduction relationships	2020	[51]
		scGNN	GNNs for representation of gene expression and cell–cell relationships	2021	[52]
	Network relationship analysis and representation	Literature co-occurrence	Co-occurrence network between syndromes-level and symptoms-level.	2014	[60]
		AMNE	Deep auto-encoder model for formula-symptom network construction	2019	[63]
		MHADTI	Heterogeneous information network embedding for drug–target interactions prediction	2022	[67]
		GLIM	Graph embedding algorithm for heterogeneous biological network construction	2022	[65]
Network target positioning	Disease/syndrome-related gene prediction	CPHER	Logistic regression-based method for disease-gene prediction	2008	[14]
		PTsGene	Network topology-based method for diseases/syndromes-genes prediction	2020	[77]
		CIPHER-SC	Graph convolution network for disease-gene prediction	2020	[78]
	Target prediction of compound in TCM	DrugCIPHER	Regression based method for compound-related targets prediction	2010	[15]
		idTRAX	ML method for effective anti-cancer drug targets	2019	[80]
		HGNA-HTI	Heterogeneous GNN for herb-target interactions prediction	2021	[82]
		DrugBAN	Bilinear attention network for drug–target prediction	2023	[87]
Network target navigating	Disease–syndrome relationships navigating	Literature co-occurrence	Relationships between syndrome-level and symptom-level.	2014	[60]
		CIPHER	Relationships between spleen qi deficiency syndrome and digestive diseases	2020	[92]
		RNA-seq, DIA-based proteomics, and untargeted metabolomics	Biological basis of PBS syndrome in CHD	2022	[42]
	Herbs/formulas–disease relationship navigating	SVM, MLR, RF and Fully Connected Neural Network	ML and deep learning methods for optimum formulas for Alzheimer’s disease	2019	[100]
		FordNet	Convolution neural network for formula recommendation with phenotype and molecule information	2021	[99]
		KDHR	Graph convolution network for herb recommendation with symptom and herb features representation	2022	[95]
	TCM formula-related MoA navigating	DMIM	Network topology method for identifying useful relationships between herbs in formulas	2010	[109]
		Random walk with restart (RWR)	Efficacy of Si Ni San (SNS) intervention in NAFLD	2021	[104]
		Graph embedding and graph convolutional network	Reusable drug combination for COVID-19 from 480 Chinese herbal medicines.	2022	[112]

### Network relationship mining

#### Network relationships mining from prior knowledges

**Figure 2:**
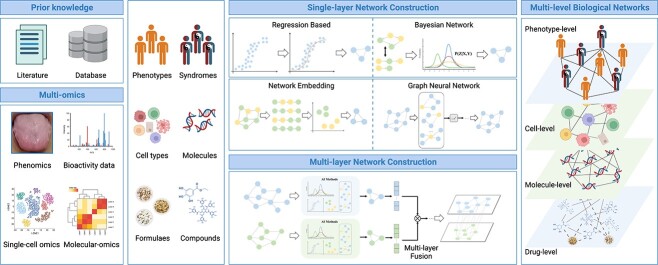
**Framework of network relationship mining.** Network relationships among phenotypes, cells, molecules and drugs, can be mined from prior knowledges, literature and omics data with methods categozied as single-layer and multilayer network construction.

With the accumulation of biomedical knowledges, AI-based mining of relationships between drugs/formulas and diseases/syndromes from prior knowledges is the premise and foundation of TCM-NP, and has emerged as a current research topic of great interest. From a network perspective, the network relationships mainly involve disease/syndrome-level, biological molecule-level and drug/formula-level (Figure 2). At the drug/formula-level, NLP techniques are mainly used to mine the relationships between different clinical symptoms related to diseases/syndromes from prior knowledges. According to the TCM clinical diagnosis and treatment theory, we could not only infer the similarities of clinical symptoms by mining clinical phenotype entries [18], but also infer the relationships between syndromes and phenotypes [19–23]. At the level of drugs/formulas, the structural similarity relationships between the components contained in formulas are mainly mined from databases such as PubChem [24], ChEMBL [25], CDCDB [26] and DrugCombDB [27], as well as the relationships between the herbs contained in formulas and their properties (e.g. relationships between Cold/Hot herbs) mined from TCM-related databases such as ETCM [28], HERB [29] and TCMBank [30]. At the molecular level, relationships such as protein–protein interactions (PPI), signal and transcriptional regulation between molecules are mainly mined from databases such as STRING [31], KEGG [32] and BioGRID [33]. The theory of TCM emphasizes ‘treatment based on syndrome differentiation’ and provides treatment plans based on patients’ different syndromes and phenotypic characteristics, so there are also relationships between syndromes/phenotypes and the efficacy of herbs/formulas, such as the relationships between syndromes and herbs/formulas mined from databases such as SymMap [34] or based on the principle of co-occurrence in literature [35]. At the same time, it is worthy of our attention for the relationships between macro- and micro-level objects. Thus, the network relationships also include those between compounds or natural products and their relevant molecular targets mined from databases such as HIT [36], TCMGeneDIT [37], DrugCentral [38] and DrugBank [39]. These network relationships mined from literature or public databases provide a biological basis for understanding TCM from a systemic perspective and laying a data foundation for conducting in-depth methodological analysis using AI methods.

#### Network relationships mining from omics data

Considering the abundant features embedded in omics data, it is possible to infer diverse relationships, especially those among micro-level elements, including cells, genes and proteins. In recent years, a large amount of omics data related to TCM have been accumulated, including bulk transcriptomics [40, 41], metabolomics [42, 43] as well as single-cell omics [44–46] related to specific herbs and/or TCM syndromes (Figure 2). From a data perspective, computational methods related to mining network relationships from omics data are similar for both TCM and Western medicine. Therefore, AI-based network relationships mining of omics data could also be widely applied in the field of TCM-NP.

Taking the transcriptomics as an example, the common method to mining potential relationships is to construct the co-expression network using canonical ML algorithms, such as statistical analysis [47] or regression [48] . For example, Li et al. [40] applied the ML-based strategy that integrated gene co-expression patterns with network topological features, to construct the molecular network underlying Cold and Hot syndrome, from which a series of syndrome-related biomarkers were identified. Furthermore, considering the noise within transcriptomics data, several studies have utilized Bayesian models to integrate omics data with prior knowledge to infer more reliable network relationships [49, 50]. For instance, Li et al. combined literature and micro-array gene-expression data to refine a gene network [49]. Greene et al. proposed a data-driven Bayesian inference framework that integrated hierarchy-aware knowledge and data compendium, to construct diverse human tissue-specific networks to understand multi-cellular function and disease [50]. In addition to gene co-expression relationships, several studies have inferred potential causal relationships from transcriptomic data by integrating gene regulatory or signal transduction relationships from prior knowledges, thus providing computational inference for identifying upstream regulatory factors [51].

With the development of single-cell sequencing technology, a large amount of single-cell transcriptomics data related to TCM have been accumulated [44–46], providing an unprecedented data resource for network relationship mining. For example, Zhang et al. [44] constructed a single-cell transcriptomics atlas from gastric premalignant and early-malignant patients with Hot syndrome. Given that drop-based single-cell sequencing technology enable to simultaneously generate transcriptomic profiles for thousands of cells, it provides an unprecedented opportunity for deep learning-based cell-level network analysis [52–56] and inferring cell-specific gene regulatory networks (GRNs) [57]. For example, Wang et al. introduced a single-cell graph neural network (scGNN) that used GNNs to establish and aggregate cell–cell relationships which provided an effective representation of gene expression and cell–cell relationships [52]. Although the current AI-based research for exploring network relationships from TCM-related omics data is still insufficient, these omics-based network mining methods will greatly expand our understanding of TCM biology at the systematic level and lay a solid foundation for the development of TCM-NP methodology.

#### Network relationship analysis and representation

Analysis and representation of multilayer network relationships are also important in network relationship mining. In this regard, AI-based methods still play an increasingly important role. In terms of disease/syndrome-related network relationship analysis, the similarity of network modules composed of different phenotypes belonging to the same syndrome or different syndromes of the same disease is evaluated by network topology-based methods [58–60], i.e. relationships between phenotype-level and disease/syndrome-level. For example, Zhou et al. [60] constructed a clinical phenotype network (CPN) to investigate the promiscuous boundary of syndromes and the co-occurrence of symptoms. In term of herb/formula-related network relationship analysis, the similarity of network modules composed of different herbs belonging to the same formula can be evaluated by integrating relationships between herb targets [61]. It is also possible to evaluate the similarity between network modules composed of formulas and the clinically intervened syndromes [62, 63]. Ruan et al. [63] proposed a novel deep auto-encoder model called AMNE to automatically detect the specific herbs for symptoms in TCM formulas. In addition, the network analysis of diseases/syndromes and herbs/formulas demonstrates that network nodes of multilevel have modular properties and that there are similar relationships between network modules of different levels. This rule is an important implication of the ‘network target’ theory and serves as the basis for the development of methodology for TCM-NP.

As the size and heterogeneity of network nodes increase, characterizing and low-dimensional representation of the network are key aspects of network relationship mining. The core of network representation is to vectorize or low dimensionally represent the network nodes, thereby analyzing the potential relationships of different network nodes from a systematic perspective. In this aspect, network embedding methods such as node2vec have become the main tools for network representation [63–71]. For example, Tian et al. [67] achieved accurate predictions of drug-targets interactions by low-dimensional representation and fusion of features. Furthermore, Hou et al. [65] integrated diseases/syndromes phenotypes, tissues, cell types and molecular interaction data and established a human multilevel heterogeneous biological network based on a graph embedding algorithm, achieving high performance in multiple tasks.

### Network target positioning

**Figure 3:**
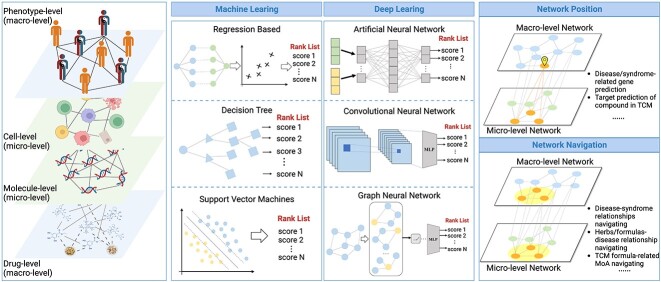
**Framework of network target analysis.** The network target positioning and navigating analysis mainly involve using classical ML-based methods and deep learning-based methods to dissect the multilayer biological networks involving phenotype, cells, molecules and drugs.

The core of AI-based network target positioning is to solve the following two important problems: (i) how to predict genes related to phenotypes/syndromes; and (ii) how to predict targets related to herbs/formulas, as shown in Figure 3.

#### Disease/syndrome-related gene prediction

The syndrome can be regarded as a personalized clinical phenotype characterization. Individualized clinical phenotype-related gene prediction is an important way to achieve personalized diagnosis and treatment. The core idea of the phenotype/syndrome-related gene prediction algorithms based on biological networks is to use the ‘multilevel modular relationship’ law and predict based on the network topology features. In this regard, traditional ML methods such as regression [14]and random walk (RW) [72, 73] were first applied to phenotypes/syndromes-gene prediction. For example, the CPHER algorithm [14] proposed by Wu et al. is considered one of the most representative algorithms [74], which provides a paradigm for research on AI algorithms related to macro- and micro-level biological networks. The CIPHER algorithm has been successfully applied to the mechanism analysis of complex diseases [40, 75, 76]. Inspired by the CIPHER algorithm, a series of network-based gene prediction algorithms for syndrome-related phenotypes or clinically relevant symptoms have been proposed. For example, PTsGene [77] combined TCM clinical phenotypes with experimental results to establish a TCM symptoms-genes dataset and achieved high-precision prediction of diseases-syndromes genes. In recent years, with the proposal of GNN learning algorithms, it has gradually become a hot topic and achieved excellent performance for considering the phenotype-molecule networks as complex graph structures for learning and inference. For example, CIPHER-SC [78] fused single-cell information to build a multilevel biological network to achieve disease gene prediction based on a graph convolution neural network. Collectively, these studies demonstrate that AI-based network analysis models could address the issue of gene predictions for TCM syndrome, paving the way to understand the biological basis of complex TCM terms.

#### Target prediction of herb compounds

Similar to genes prediction of diseases/syndromes, it is also a prerequisite to revealing the pharmacology of TCM through accurately predicting the related targets of herbs/formulas and their compounds. From the methodological perspective, the prediction of compound-related targets can be divided into traditional ML prediction methods based on network topology analysis (e.g. regression) [15–81] and prediction methods based on deep learning models [64, 69, 82–89]. Among them, the drug-target prediction algorithm DrugCIPHER [15] proposed by Zhao et al. is a representative algorithm that applies network-based regression analysis to achieve high-precision prediction of compound-related targets. In addition, genomics data have also been integrated into the network-based analysis to achieve high-precision predictions [80] [81]. Compared with traditional ML methods, deep learning models can integrate a large amount of heterogeneous information such as drug similarity structure, treatment information of diseases and drug activity to conduct more complex drug-targets prediction tasks while improving prediction accuracy [64, 69, 82, 85–89].

### Network target navigating

TCM has the significant characteristics of ‘multiple components, multiple targets and systemic regulation.’ Another key challenge in deciphering the biological basis of TCM is how to understand the relationships between various TCM terms from the perspective of biological networks, i.e. network target navigating, including the disease–syndrome relationships, herb/formula–disease relationships, the compatibility rules of formulas and the synergy rules of their components. In this regard, it is an important methodology for the network modularity analysis, where traditional ML and deep learning models play an important role (Figure 3).

#### Disease-syndrome relationships navigating

Both phenotypes and syndromes are clinical description of the complex human body. From a macro perspective, by analyzing the network topology similarity between disease-related phenotypes and syndrome-related clinical features, the relationships between diseases and syndromes can be revealed [60, 90]. In this regard, Zhou et al. [60] established a CPN, providing a basis for personalized diagnosis and treatment of TCM. From a micro perspective, we can achieve the integration of disease diagnosis and syndrome differentiation by performing relevant gene prediction or omics data analysis based on the disease phenotypes and syndrome characteristics, followed by the construction of similarity metrics and correlation analysis based on biological molecular networks [40, 42, 65, 91–93, 94]. For example, mining the relationships between Cold/Hot syndromes and neuro-endocrine-immune (NEI) biological networks [91], analyzing the biological basis of spleen deficiency syndrome and its relationships with digestive diseases [92], analyzing the biological basis of PBS syndrome in coronary heart disease (CHD) and establishing diagnostic markers to achieve ‘same disease with different treatments’ [42].

#### Herb/formula–disease relationship navigating

Navigating the herb/formula–disease relationships in TCM-NP mainly refers to network-based precision recommendation of herb/formulas in the context of specific diseases or syndromes. Usually, the AI-based formula recommendation methods infer relationships between herbs/formulas modules and specific symptoms/syndromes based on the macro-level information from prior knowledges [95–98]. With the accumulation of multi-omics and micro-level network analysis, more accurate recommendation methods have been proposed by integrating herbs/formulas-target network and disease/syndrome-gene network. For example, Zhou et al. proposed an intelligent formula recommendation system FordNet [99] integrating multilevel information, which can be considered one of the representative works of integrating macro and micro information. Moreover, new indications of TCM formulas could also been discovered through inter-module association analysis based on the herbs/formulas-target network and the disease/syndrome-gene network [100–103]. For example, Chen et al. [100] utilized a NP-based approach to investigate TCM candidates that can dock well with multiple targets and successfully found the optimal TCM formula for treatment of Alzheimer’s disease.

#### TCM formula-related MoA navigating

The general research framework for the TCM formula-related MoA navigating with TCM-NP includes: identification of active ingredients from the TCM formula; construction of the network underlying the disease/syndrome that TCM formula intervenes; discovery of the network target of the active ingredients of the TCM formula based on the disease/syndrome-related network. To date, this framework has been widely applied in the MoA analysis of many formulas [104–107]. Moreover, studying the compatibility law of herbs/formulas is also an important issue in TCM-NP. The essence of studying the compatibility law of formulas is also to explore the modular law of component–target relationships, and to further establish the association rules of formulas by identifying the herb–herb relationships [108–112]. Li et al. [109] established a distance-based mutual information model (DMIM) to measure the interaction between herbs and constructed a herb network, thus identifying useful relationships between herbs in many formulas. In addition, Yang et al. [112] discovered a reusable drug combination for COVID-19 from 480 Chinese herbal medicines. In summary, analyzing the herb–herb relationships between herbs/formulas modules is achieved by mining the MoA of multiple components in herbs/formulas, as well as through the application of AI methods for mining the compatibility rules of formulas.

## REPRESENTATIVE APPLICATION OF TCM-NP IN COLD/HOT SYNDROMES

**Figure 4:**
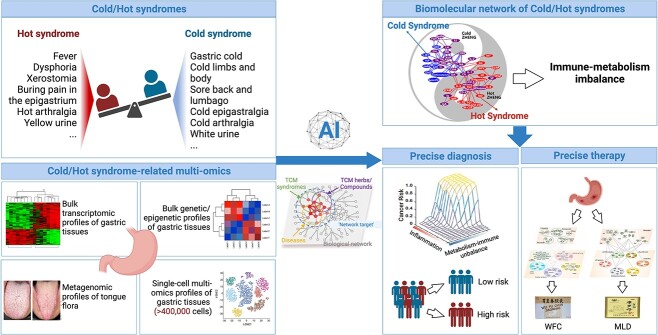
**Construction, analysis and application of TCM-NP in Cold/Hot syndromes and herbs.** With collection and dissection of Cold/Hot syndrome-related multi-omics data, the Cold/Hot syndrome-related molecular network has been firstly constructed to facilitate the precise diagnosis and therapy for gastric diseases [113, 115, 116].

Cold/Hot syndromes are fundamental concepts in TCM, which has been widely applied in personalized clinical practice. In this section, we would present the representative application of TCM-NP in Cold/Hot syndromes, where we would emphasize the biological basis of Cold/Hot syndromes and its potential in precision diagnosis and treatment of diseases, as shown in Figure 4.

Cold/Hot syndromes are characterized by a series of clinical features in patients. Revealing syndrome-related biological basis involves understanding the micro-level biological molecules related to macro-level clinical features. As early as 2007, Li et al. firstly utilized literature mining and network topology analysis methods to construct the molecular network underlying Cold/Hot syndromes in the context of the NEI system [91]. It revealed that the Cold syndrome-related sub-network was primarily characterized by hormone-related factors, whereas the Hot syndrome-related sub-network was dominated by immune-related factors, and these two networks are connected by neuro-transmitters. This study created a precedent to reveal biological basis of TCM syndromes by using TCM-NP approaches. Subsequently, by integration of the network target positioning algorithm CIPHER [14] and Cold/Hot syndrome-related transcriptomics data, Li et al revealed that the Cold/Hot syndromes involved the network imbalance underlying metabolism-immune regulation and identified syndrome-related biomarkers, including Cold syndrome-related ones, such as LEP and NOS1, and Hot syndrome-related ones, such as CCL2 [40]. Thus, these findings indicated that the issue of the biological basis of Cold/Hot syndromes could be addressed with TCM-NP methodologies.

Then, network target navigating analysis could be used to understand the relationships between Cold/Hot syndrome-related genes and the occurrence and development of diseases, thereby guiding individualized disease diagnosis and treatment. Specifically, network target navigating analysis based on network topology can be used to analyze the network relationships between Cold/Hot syndrome-related genes and disease-related molecules, from which network modules could be identified and further analyze their clinical value [113]. Then, a deep learning model was established, which indicated that the tongue coating image features related to Cold/Hot syndromes exhibited predictive value for early gastric cancer risk [114]. In addition, Cold/Hot syndrome-related network nodes might show associations with tumor development and progression. For example, by integrating network-based prediction with omics data, the pancreatic cancer prognosis-related biological network has been established, where several nodes involved in Hot-related TGFbeta signaling pathways have been uncovered [75]. Thus, these studies highlight the important role of understanding Cold/Hot syndromes in facilitating disease precision diagnosis and treatment, by combining TCM-NP-based computational analysis with biological and clinical experiments.

Another important issue of revealing the biological network basis of Cold/Hot syndromes is to guide the precise use of TCM formulas. According to the principle of syndrome differentiation and treatment, the use of TCM formulas in different populations should be based on the patient’s personal syndromes and clinical characteristics. Therefore, through network target navigating analysis, which is the analysis of the topological relationships between the molecular network related to Cold/Hot syndromes and the target networks of Cold/Hot herbs, we can reveal the MoA of different Cold/Hot herbs and guide their precise use. As expected, through network topology analysis, we found that the Cold syndrome-related herbs tended to be enriched in the Hot syndrome-related network modules, while the Hot syndrome-related herbs tended to be enriched in the Cold syndrome-related network modules [91]. This distribution pattern is consistent with the principle of ‘Warming the Cold and Cooling the Hot’ in TCM. In addition, combining network analysis with experimental verification can also reveal the pharmacological mechanisms of formulas, guiding individualized treatment for patients. For example, based on gastritis Cold/Hot syndrome-related biological network, we uncovered different MoAs for two representative TCM formulas for the treatment of gastritis, WeiFuChun (WFC) and MoLuoDan (MLD)，where WFC [115] focused on regulating inflammatory pathways, while MLD [116] focused on inhibiting fatty acid metabolism, which would aid precision treatment of gastritis in clinical practice.

## CONCLUDING REMARKS AND FUTURE PERSPECTIVES

TCM emphasizes treatment based on syndrome differentiation and the usage of formulas, and has the characteristics of holistic regulation. TCM-NP, as an emerging interdisciplinary discipline, has been widely accepted in TCM research and has been widely used in the research of various traditional medicines around the world. In the era of AI algorithms and multi-omics, TCM-NP has ushered in new development opportunities and is expected to lead to some breakthroughs.

Importantly, we would present several profound and insightful perspectives for the development of TCM-NP methodologies in the era of AI and multi-omics technologies, including: (i) Network-based integration with AI techniques; (ii) Deep network relationships mining; (iii) Network target quantitative positioning and navigating; (iv) Developing deep interpretable network pharmacological models.

Firstly, the integration of multi-modal data with AI techniques is an important research direction and hotspot in the NP methodologies. In summary, AI-based network integration analysis mainly includes the following three aspects: integration of multi-omics data, integration of omics data with prior knowledge and integration of multi-source heterogeneous biological networks. As for the first aspect, it mainly involves using biological networks to mathematically represent multilevel information within omics data, such as cell–cell, cell–molecule and molecule–molecule relationships. For example, the Scissor algorithm developed by Sun et al. [117] could infer the ‘phenotype-cell’ relationships by proposing a network regularized sparse regression model on the correlation matrix that integrated phenotype-associated bulk and single-cell expression data. In the future, we could expect the emergence of TCM-related integration algorithms with the accumulation of multi-omics data. As for the second aspect, it is pivotal to perform network-based integration of prior knowledge with omics data to reduce the effects of noise and limited samples of omics data. In this regard, Bayesian network is the commonly-used AI model that usually views the knowledge-derived relationships as prior distributions, while those omics-derived ones as posteriors. For example, Greene et al. [50] utilized the Bayesian model to construct a tissue-specific network with integrating tissue-level relationships from the Gene Ontology database and co-expression ones from transcriptomic data. In recent years, several deep learning models, including transfer learning, have been employed to integrate relationships between prior knowledge and omics data, such as Geneformer [11]. As for the third aspect, although different biological networks may represent heterogeneous information, they can be mathematically modeled as adjacency matrices, and thus could be integrated under unified AI algorithms, which provides the possibility to integrate heterogeneous biological networks from the NP perspective.

Secondly, Molecules are typically expressed in a tissue-specific and even a cell type-specific manner, which demonstrates the pivotal role of construction of tissues/cells-specific networks in elucidating mechanisms of syndromes/diseases as well as MoA of herbs/formulas. Thus, new AI methods are needed to systematically mine the tissue-level and/or cellular-level relationships between phenotypes and molecules [118, 119]. For example, Chen et al. [118] proposed a transfer learning framework to predict cancer drug response at the single-cell level. scDEAL could infer cell-specific network and interpret features related to drug resistance by using the transfer learning models pre-trained on the collected bulk and single-cell RNA-seq data. We hope that using AI methods to deeply mine relationships between cells and cell-type specific molecules with diseases/syndromes or formula/herb intervention-related single-cell multi-omics data would be a new perspective for understanding the MoA of TCM in NP.

Thirdly, we are keenly aware of the inevitability of the network target mode moving toward quantitative research. Thus, it is essential for TCM-NP to design quantitative indicators to measure network target effects, including intervention dosages and drug responses, and then propose AI models to quantitatively predict network targets from the established network. In this regard, the quantitative intervention model of drug combinations on the diseases/syndromes biological network proposed early as 2015 [120] might pay way for the development of new quantitative AI models.

Finally, traditional AI models are usually hampered by the ‘large scale, diverse modalities’ network data and model interpretability. Therefore, we emphasize the importance of developing deep interpretable network relationship inference frameworks, which aim to uncover network relationships among features derived from multi-omics and multi-modal biological data and identify the key network modules by integrating both macro- and micro-level information [10]. The deep interpretable network relationship inference framework offers two main advantages: (i) the network hierarchy and nodes in the model possess clear biological implications, allowing for interpretable feature learning, and (ii) the model not only learns the features of the network but also their associations, thereby expanding the feature space and facilitating the low generalization and insufficient interpretability problems of AI-based methods in biological network. Moreover, the rapid advancement of Language Model (LM) technologies, such as Large Language Models (LLMs), has overcome the limitations of computational resources and data volume, which might help develop deep interpretable network relationship inference models and facilitate the development of methodologies in NP.

**Key Points**

- We give an overview of the origin and development of TCM-NP, and explain in detail the core theories and methods of TCM-NP, especially the analysis of network targets by integrating AI methods and multi-omics sequencing technology;
- We summarize TCM-NP-involved AI methods into three categories: network relationship mining, network target positioning and network target navigating, where representative methodological cases are introduced in each category.
- We present the typical application of TCM-NP in Cold/Hot syndromes (a.k.a. TCM ZHENG), two basic concepts of TCM, for dissecting network relationships and their clinical value among syndromes, diseases and formulas.

## FUNDING

This work is supported by research grants from the National Natural Science Foundation of China [T2341008, 82305047] and sponsored by Tsinghua-Toyota Joint Research Fund, and Anhui Province Traditional Chinese Medicine Science and Technology Research Project [202303a07020001].

## DATA AVAILABILITY

All data used in this review is publicly available. All the figures in this review were created with BioRender.com.

## Author Biographies

**Peng Zhang** is an assistant research fellow at Tsinghua University. His research focuses on the development of network pharmacology and multi-omics integration methods for cancer precision prevention and TCM modernization.

**Wuai Zhou** is an expert at China Mobile Information System Integration Co, Ltd. His research focuses on the development of artificial intelligence/machine learning method and its application in the field of network pharmacology/digital government.

**Dingfan Zhang, Lan Wang, Boyang Wang and Tingyu Zhang** are PhD students in the field of network pharmacology with a background in automation engineering focusing on the development and application of computational methods to study the multilevel relationships of biological systems.

**Shao Li** is a tenured professor at Tsinghua University, and the Fellow of the Royal Society of Chemistry/Biology. He has made great contributions to establishing network pharmacology theory and methodologies to promote traditional medicine development and cancer prevention.
